# New Insights into the Genomic Structure of *Avena* L.: Comparison of the Divergence of A-Genome and One C-Genome Oat Species

**DOI:** 10.3390/plants11091103

**Published:** 2022-04-19

**Authors:** Alexander A. Gnutikov, Nikolai N. Nosov, Igor G. Loskutov, Elena V. Blinova, Viktoria S. Shneyer, Nina S. Probatova, Alexander V. Rodionov

**Affiliations:** 1 Department of Genetic Resources of Oat, Barley, Rye, Federal Research Center N. I. Vavilov All-Russian Institute of Plant Genetic Resources (VIR), 190000 St. Petersburg, Russia; a.gnutikov@vir.nw.ru (A.A.G.); i.loskutov@vir.nw.ru (I.G.L.); e.blinova@vir.nw.ru (E.V.B.); 2 Laboratory of Biosystematics and Cytology, Komarov Botanical Institute of the Russian Academy of Sciences, 197376 St. Petersburg, Russia; shneyer@rambler.ru (V.S.S.); arodionov@binran.ru (A.V.R.); 3 Laboratory of Botany, Federal Scientific Center of the East Asia Terrestrial Biodiversity, Far Eastern Branch of the Russian Academy of Sciences, 690022 Vladivostok, Russia; probatova@ibss.dvo.ru

**Keywords:** rRNA genes, grasses, hybridization, molecular phylogeny, next-generation sequencing, rDNA

## Abstract

We used next-generation sequencing analysis of the 3′-part of 18S rDNA, ITS1, and a 5′-part of the 5.8S rDNA region to understand genetic variation among seven diploid A-genome *Avena* species. We used 4–49 accessions per species that represented the As genome (*A. atlantica*, *A. hirtula*, and *wiestii*), Ac genome (*A.* *canariensis*), Ad genome (*A. damascena*), Al genome (*A. longiglumis*), and Ap genome (*A. prostrata*). We also took into our analysis one C-genome species, *A. clauda*, which previously was found to be related to A-genome species. The sequences of 169 accessions revealed 156 haplotypes of which seven haplotypes were shared by two to five species. We found 16 ribotypes that consisted of a unique sequence with a characteristic pattern of single nucleotide polymorphisms and deletions. The number of ribotypes per species varied from one in *A. longiglumis* to four in *A. wiestii*. Although most ribotypes were species-specific, we found two ribotypes shared by three species (one for *A. damascena*, *A. hirtula*, and *A. wiestii*, and the second for *A. longiglumis*, *A. atlantica*, and *A. wiestii*), and a third ribotype shared between *A. atlantica* and *A. wiestii*. A characteristic feature of the *A. clauda* ribotype, a diploid C-genome species, is that two different families of ribotypes have been found in this species. Some of these ribotypes are characteristic of Cc-genome species, whereas others are closely related to As-genome ribotypes. This means that *A. clauda* can be a hybrid between As- and C-genome oats.

## 1. Introduction

The genus *Avena* L. belongs to the subtribe *Aveninae* J. Presl, tribe Poeae (=Aveneae chloroplast group) of the family Poaceae Barnhart [1,2]. Currently, the genus comprises 12 diploid (2*n* = 14), 8 tetraploid (2*n* = 28), and 6 hexaploid (2*n* = 42) species [3]. With a few exceptions, the oat species are annual and self-pollinated. Cytogenetic and genomic studies assigned the different *Avena* species into A, B, C, and D genomes. The A and C genomes differ from each other significantly, while the B and D genomes are derivates of the A genome [4,5,6,7,8,9,10,11,12]. The estimated time of divergence of the A/B/D genomes from the C genome is contradictory in the literature, which includes 5–13 million years ago (MYA) [13], 8–9 MYA [14], 20 MYA [15], or 25 MYA [16]. Such discrepancies in the divergence time between the A/B/D and C genomes are likely due to difficulties in normalizing the chronological scale of the macrofossil remains in Poaceae [17,18,19]. In addition, timescale calibration is complicated due to an unusually low rate of nucleotide substitutions observed in Poaceae [13,18].

Chromosome sets of *A. canariensis* B.R. Baum, Rajhathy, and D.R. Sampson (Ac), *A. damascena* Rajhathy and B.R. Baum (Ad), *A. longiglumis* Durieu (Al), and *A. prostrata* Ladiz. (Ap), are species-specific and differ from each other in the number of acrocentric chromosomes. The As genome variety has been found in several diploid species: *A. atlantica* B.R. Baum and Fedak, *A. hirtula* Lag., *A. strigosa* Schreb., *A. wiestii* Steud. [3,4]. Species with different A genomes do not naturally interbreed. Hybrids can be produced by artificially crossing a pair of A genome species, but the offspring are usually sterile, although some studies reported fertile offspring [20,21,22,23]. It was shown that the A genome of diploid *Avena* species retains a remarkable degree of synteny in comparison with that of barley, while C-genome diploids have undergone a relatively greater degree of chromosomal rearrangement, suggesting the presence of underlying genomic instability [13].

Phylogenetic analyses conducted using random amplified polymorphic DNA (RAPD) and restriction fragment length polymorphism (RFLP) markers revealed two groups for the A-genome diploid *Avena* species that correspond to As-genome species and all other species with Al, Ad, and Ac genomes [24]. These results were confirmed in a recent study by Maughan and co-workers [13] using 7221 single nucleotide polymorphisms (SNPs). One of the two distinct clades for the A-genome diploids species consisted of primarily accessions in the As subgenome (*A. atlantica*, *A. hirtula*, the domesticated forms of *A. strigosa*, and *A. wiestii*). The second clade comprised accessions from the *A. canariensis* (Ac), *A. damascena* (Ad), and *A. longiglumis* (Al). In a study conducted using 12,672 chloroplast and mitochondrial SNPs, the divergence time between the As genome and Al genome was estimated to be about 11 MYA, between the Al branch and the branch of Ad + Ac genomes, about 13 MYA [15].

One of the approaches to study the origin and relationship among species can be the study of intragenomic rDNA polymorphism [25,26,27]. The 35S (in plants and yeasts) and 45S (in animals) rRNA genes encoding 18S, 5.8S, and 26S rRNA are essential constituents of all eukaryotic genomes [28,29,30]. Plants are known to bear a high number of the 35S rRNA genes in the haploid genome, which range from 200 to 22,000 (>2500 on average) that are arranged in tandem arrays on one or several chromosomes [30,31]. In all studied A-genome *Avena* diploids species, two to three 35S rDNA loci per haploid genome have been reported [7,32,33,34].

The ideas on the role and place of polyploidy in processes of progressive plant evolution have been recently drastically revised. According to the data of comparative genomics, whole-genome duplication processes that usually indicate hybridization events occurred in all phylogenetic branches of the land plants [35]. Previously, the hybrid origin of a species or a particular plant could only be suggested by botanists based on taxonomically significant characters (trait) in one plant. Later, researchers began to detect hybrid taxa and found different positions of the taxon on the phylogenetic trees that were built using chloroplast (maternally inherited) and nuclear (biparental inherited) markers. Additionally, methods of marker region cloning yielded significant results in determining parental taxa in the case of stabilized hybrid species [36]. Locus-specified next-generation sequencing can reveal hidden hybridization even when morphological features are stable and do not show on hybrid origin [37]. We need to notice that next-generation sequencing methods allow us to obtain a minor quantity of marker sequences that cannot be revealed by the Sanger method and cloning [38].

We took the ITS1–5.8S rDNA region into our analysis as a marker sequence. Some previous research found that the ITS1 region in many cases is a better DNA barcode marker than ITS2 [39] because of the higher substitution rate and less conservative structure [40,41,42]. Recently, while studying the intragenomic polymorphism of rDNA of *Avena* species with C genomes, we suggested that the diploid species *A. bruhnsiana* and *A. clauda* are homoploid hybrids [43]. The objectives of the present study were to understand the genetic variation among seven A-genome *Avena* species that represented five subgenomes (Ac, Ad, Al, Ap, and As) and detect the relationship of A-genome ribotypes found in C-genome species *A. clauda* using 18S rDNA, ITS1, and 5.8S rDNA sequences.

## 2. Materials and Methods

The present study was conducted on a total of 169 accessions from seven oat species (Table 1) that represent the As-genome (*A. atlantica*, *A. hirtula*, and *wiestii*), Ac-genome (*A. canariensis)*, Ad-genome (*A. damascena*), Al-genome (*A. longiglumis*), Ap-genome (*A. prostrata*), and one Cc-genome species, *A. clauda*, in which we previously found the sequences related to the A genome. The number of accessions per species varied from 4 in *A. damascena* to 49 in *A. hirtula.* All samples were obtained from the Federal Research Center N. I. Vavilov All-Russian Institute of Plant Genetic Resources (VIR).

Genomic DNA was extracted from seeds using a Qiagen Plant Mini Kit (Qiagen Inc., Hilden, Germany) according to the instruction manual. For next-generation sequencing, we used the 3′-part of 18S rDNA (70 bp), the complete ITS1 (228 bp), and a 5′-part of the 5.8S rDNA region (53 bp). The fragments were amplified and sequenced at the Center for Shared Use “Genomic Technologies, Proteomics and Cell Biology” of the All-Russian Research Institute of Agricultural Microbiology on an Illumina Platform MiSeq. PCR was carried out in 15 µL of the reaction mixture containing 0.5–1 unit of activity of Q5^®^ High-Fidelity DNA Polymerase (NEB, Ipswich, MA, USA), 5 pM of forward and reverse primers, 10 ng of DNA template, and 2 nM of each dNTP (Life Technologies, ThermoScientific, Waltham, MA, USA). The PCRs for all fragments were performed using ITS 1P [44] and ITS 2 [45] primers as follows: initial denaturation 94 °C for 1 min, followed by 25 cycles of 94 °C for 30 s, 55 °C for 30 s, 72 °C for 30 s, and a final elongation for 5 min. PCR products were purified according to the Illumina recommended method using AMPureXP (Beckman Coulter, Indianapolis, IN, USA). Further preparation of the libraries was carried out in accordance with the manufacturer’s MiSeq Reagent Kit Preparation Guide (Illumina) (http://web.uri.edu/gsc/files/16s-metagenomic-library-prep-guide-15044223-b.pdf (accessed on 11 May 2020)). The libraries were sequenced according to the manufacturer’s instructions on an Illumina MiSeq instrument (Illumina, San Diego, CA, USA) using a MiSeq^®^ ReagentKit v3 (600 cycle) with double-sided reading (2 × 300 n). Sequences were pair-ended. The sequences were trimmed with Trimmomatic [46] included in Unipro Ugene [47] using the following parameters: PE reads, sliding window trimming with size 4 and quality threshold 12, minimal read length 130. Then, paired sequences were combined using the fastq-join program [48]. For our analysis, the whole massive of the sequences was dereplicated and sorted into the ribotypes with the aid of vsearch 2.7.1 [49]. The resulting sequences represent ribotypes in the whole pool of genomic rDNA, which were filtered based on their frequencies. For our analysis, we established a threshold of 20 sequences per pool of reads. The sequences were aligned using MEGA X [50], a haplotype network was built in TCS 2.1 [51] and visualized in TCS BU [52] as described in our previous study [43].

We computed the genetic distance between pairs of accessions using the Maximum Composite Likelihood algorithm and used the distance matrix to construct the neighbor-joining tree in MEGA X. We also computed the number of polymorphic (segregating) sites, nucleotide diversity (θ and π), and the Tajima test statistic (D) using the Tajima’s Test of Neutrality implemented in MEGA X. We then used the pairwise genetic distance matrix as an input file to compute the first five components in DARWin v6 [53]. The first three axes from multidimensional scaling were plotted for visual examination of the clustering patterns of accessions belonging to the different species in CurlyWhirly v1.19.09.04 (The James Hutton Institute, Information & Computational Sciences). The number of haplotypes, fixation index (F_ST_), and analysis of molecular variance (AMOVA) were computed in Arlequin v.3.5.2.2 [54].

## 3. Results

### 3.1. Sequence Variation and Ribotypes Network

**Figure 1:**
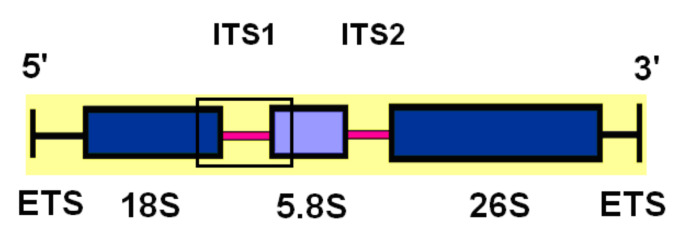
rDNA cluster and studied region (showed by a rectangle).

The aligned 3′-part of 18S rDNA, the complete internal transcribed spacer ITS1, and a 5′-part of the 5.8S rDNA sequences were 342 bp (Table S1). The studied region is presented in Figure 1.

The aligned sequences were sorted into ribotypes, with each ribotype corresponding to a unique sequence with a characteristic pattern of SNPs and deletions. Table 1 summarizes the 16 major ribotypes that were represented in the genome by more than 1000 reads. 

**Figure 2:**
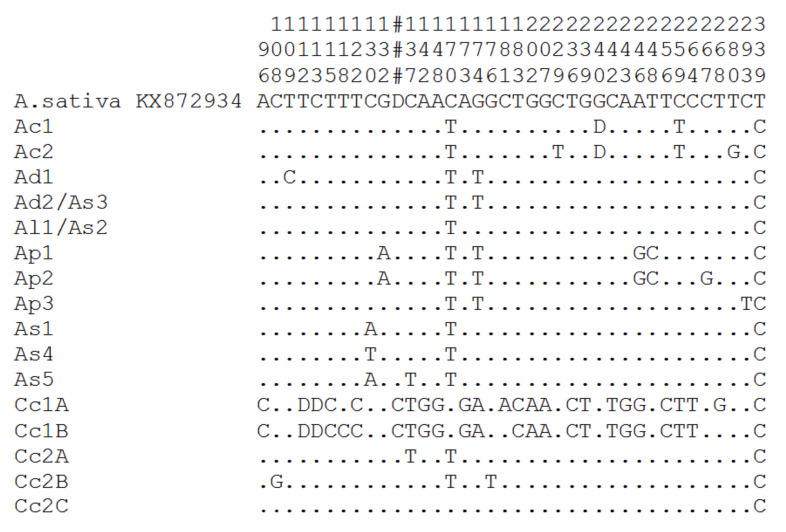
Variable sites of major ribotypes of the A-genome diploid *Avena* species: SNPs and a single nucleotide deletion. Positions with SNPs are indicated by a number. Dots indicate the identity of similar nucleotide bases with the reference sequence *A. sativa* KX872934 (A genome from the hexaploid). Position 1 of our alignment corresponded to nucleotide 218 of the reference sequence. D—deletion. #—single nucleotide deletion in the reference sequence between nucleotide 351 and 352.

We compared each of the 16 ribotype consensus 18S–5.8S rDNA–ITS2 sequences of the A-genome with the *A. sativa* A-genome sequence (Genbank number KX872934). The 18S rDNA and 5.8S rDNA had very few deviations from the consensus *A. sativa* sequence (Figure 2), which included changes from C→A or T, G→A or T (Figure 2). Overall, we found 14 variable positions with SNPs and two deletions, which suggested the presence of higher intragenomic ITS1 sequences variation in the diploid oat species.

Of the 16 ribotypes identified in the present study (Table 1), *A. longiglumis* had a single ribotype (Al1/As2). Four species had two major ribotypes each, which included Ac1 and Ac2 in *A. canariensis*, Ad1 and Ad2/As3 in *A. damascena*, As1 and Al1/As2 in *A. atlantica*, and As5 and Ad2/As3 in *A. hirtula.* Both *A. prostrata* and *A. wiestii* had more variable ITS1 patterns. We found three ribotypes (Ap1, Ap2, and Ap3) in *A. prostrata* and four ribotypes (As1, Al1/As2, Ad2/As3, and As4) in *A. wiestii.* Most ribotypes were species-specific, but some were observed in two to five species. The latter included Ad2/As3 that was observed in *A. damascena*, *A. hirtula*, and *A. wiestii,* Al1/As2 observed in *A. longiglumis*, *A. atlantica*, and *A. wiestii*, and As1 observed in both *A. atlantica*, and *A. wiestii*.

**Figure 3:**
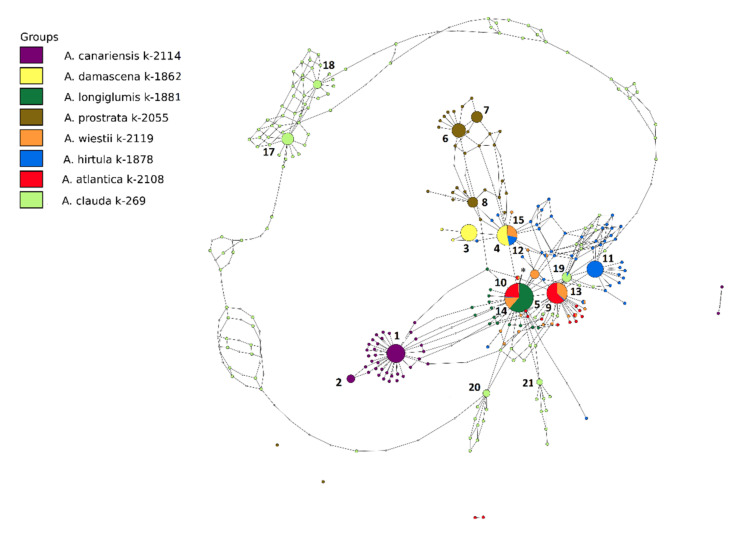
Comparisons of 16 ribotypes (#1–16) network from seven diploid A-genome *Avena* species with five ribotypes (#17–21) in a C-genome species (*A. clauda*) based on ITS1 sequences. The five *A. clauda* ribotypes were included for comparison purposes and because it had some A-genome ribotypes in its rDNA. For each ribotype, the size of the circles represents the percentage of reads as shown in Table 1. The smallest circles correspond to ITS1 variants that have been read fewer than 1000 times. See Figure 2 for details of each ribotype.

Statistical parsimony ribotype network revealed by TCS 1.21 software [51] and tcsBU [52] is presented in Figure 3. It includes 16 major ribotypes and all minor derivates of the major ribotypes that have been read more than 20 times. Minor ribotypes differing from major ribotypes by 1–3 nucleotide substitutions can be the result of mutation instability of the major ribotypes. In the network, there were two superfamilies of ribotypes: ribotypes of *A. clauda* C genome and all other ribotypes belonging to A genome. The first group was found earlier in *A. pilosa* and *A. bruhnsiana*, both species with C genomes [43]. All other ribotypes were found in the A-genome species. One can see that *A. canariensis*, *A. prostrata*, and *A. clauda* had a species-specific pattern of ribotypes. Moreover, the Clauda ribotypes Cc2A–Cc2C (##19–21 on Figure 3) were closely related to the ribotypes of species with Al and As genomes (##5, 9, 10, 13, 14 in Figure 3).

The A-genome oats exhibited a common ribotype network where some ribotypes were shared between different species, which included ribotypes Ad2/As3 (#4, 12, and 15), Al1/As2 (#5, 10, and 14), and As1 (#9 and 13). The most abundant ribotype of *A. longiglumis* Al1/As2 (9412 reads, 74% of the gene pool) was shared with the second variant of *A. atlantica* (4571 reads, 28%) and *A. wiestii* (2901 reads, 17%). The As1-ribotype of *A. atlantica* (6353, 39%) was also found in *A. wiestii* (4005 reads, 24%).

The three ribotypes in the Ap-genome *A. prostrata* species (#6-8 for Ap1, Ap2, and Ap3) and the two ribotypes in the *A. canariensis* (#1 and 2 for Ac1 and Ac2) appeared to be quite different from all other ribotypes of the A-genome species. The most frequent ribotype of *A. damascena* was also unique (#3 or Ad1, 4910 reads, 40%). The second ribotype in *A. damascena* (Ad2/As3 or #4) was shared with two As-genome species: *A. hirtula* (3587 reads, 11%) and *A. wiestii* (2598 reads, 16%). *A. hirtula* had another unique major ribotype (As5 or #11 with 12605 reads that accounted for 40% of the genome).

NGS analysis of *A. canariensis* showed that there were two close but not identical major ribotypes in its rDNA pool; however, at the same time, its genome contained minor quantity rDNA sequences of the ribotype Ad2/Al1 (37 reads) (Figure 3).

### 3.2. Genetic Diversity and Relationship

**Figure 4:**
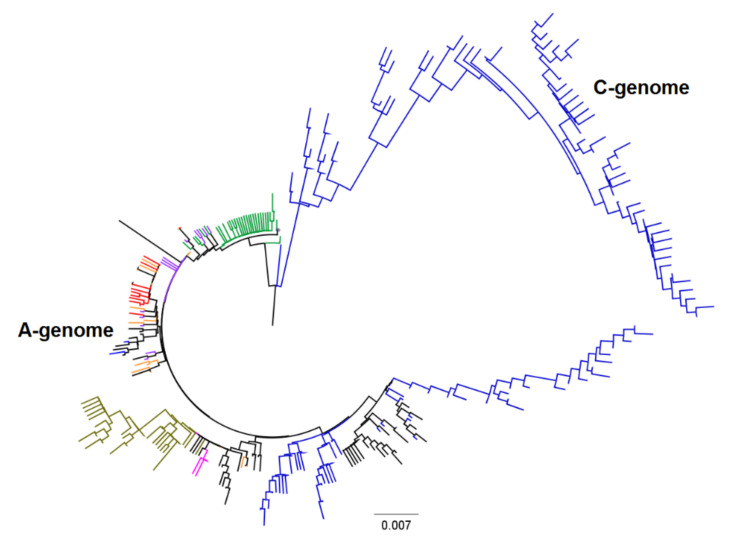
Neighbor-joining tree based on sequences of 296 accessions of A-genome oats and *Avena clauda* (C genome). The phylogenetic tree was constructed in MEGA X using the pairwise genetic distance matrix, Maximum Composite Likelihood algorithm. Font colors are as follows: *A. canariensis* (green), *A. damascena* (pink), *A. longiglumis* (purple), *A. prostrata* (olive), *A. atlantica* (red), *A. hirtula* (black), *A. wiestii* (orange), and *A. clauda* (blue).

**Figure 5:**
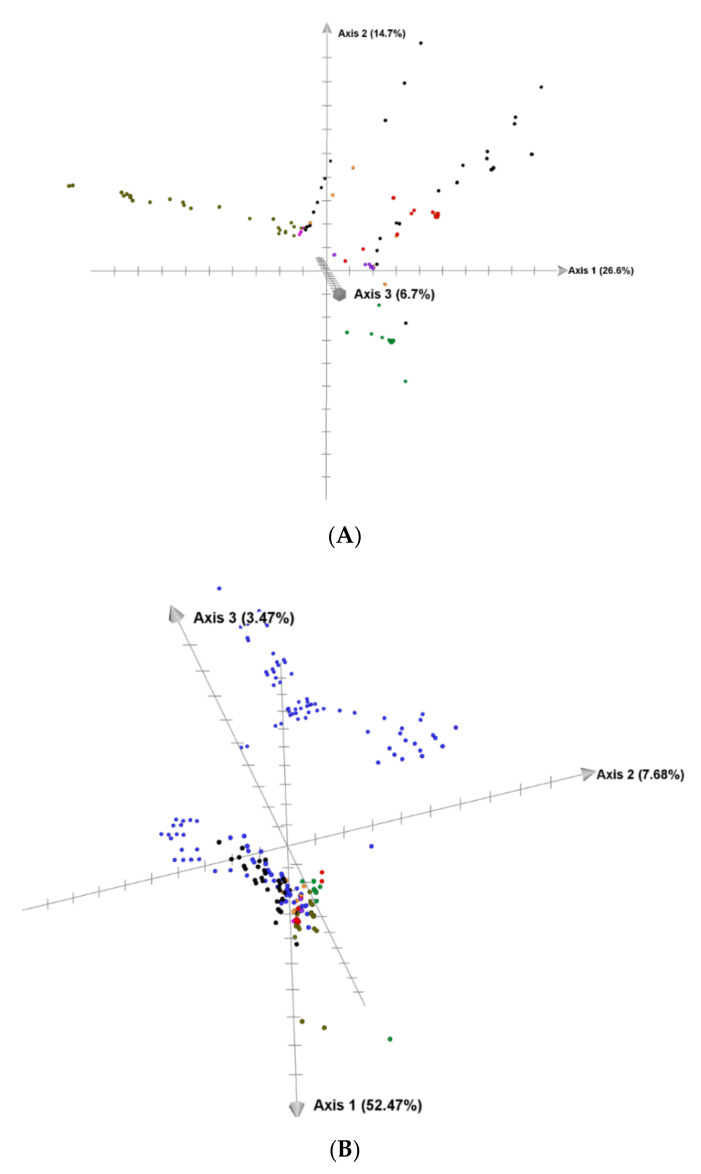
(**A**). Multidimensional scaling based on sequences of 165 of 169 accessions from seven *Avena* species. Each species was represented by 4–49 accessions. Two outlier accessions in *A. canariensis* (OM004573 and OM004575) and *A. prostrata* (OM004633 and OM004639) were found to be quite different from all other accessions in the phylogenetic tree and multidimensional scaling analysis (Figure S1) and are excluded here. The multidimensional scaling plot was plotted using CurlyWhirly v1.19.09.04. (**B**). Multidimensional scaling based on 296 accessions of eight oat species—A-genome oats and *A. clauda* (C genome). Font colors are as follows: *A. canariensis* (green), *A. damascena* (pink), *A. longiglumis* (purple), *A. prostrata* (olive), *A. atlantica* (red), *A. hirtula* (black), *A. wiestii* (orange), and *A. clauda* (blue).

Table 2 summarizes the number of sequences (S), the proportion of segregating sites (Ps), theta (ϴ), nucleotide diversity (π), and D statistics. The Ps, θ, π, and D statistics computed from all 169 accessions were 0.269, 0.047, 0.013, and −2.291, respectively. Adding *A. clauda* as the species with both (A and C) variants of the genome changed parameters to 0.318, 0.051, 0.031, and −1.154. We then compared each parameter for each species, which provided highly variable results. The highest number of sequences, segregating sites, and θ were observed within *A. canariensis*, followed by *A. prostrata*, which was due to the presence of two outlier accessions in each species. Both OM004573 and OM004575 in *A. canariensis* and OM004633 and OM004639 in *A. prostrata* were found to be quite different from all other accessions in the phylogenetic tree and multidimensional scaling (Figure S1, Figure 3 and Figure 4). The diversity indices and genetic relations among accessions and species showed different patterns when these four-outlier accessions were excluded from the analyses (Table 2 and Figure 5). For example, the ϴ values computed from 38 *A. canariensis* and 31 *A. prostrata* accessions were 0.028 and 0.025, respectively, which dropped to 0.020 and 0.012 when the two outlier accessions were excluded from each species. The four accessions may have been unique as compared with the remaining 165 accessions, but we could not rule out sequencing errors on those accessions. *A. clauda* (having both variants of the genome according to NGS data) showed a ϴ value of 0.018, and nucleotide diversity (π) was 0.037. Pairwise genetic distance computed between the 169 and 165 accessions varied from 0 to 0.082 and from 0 to 0.033, respectively (Table S2). Adding *A. clauda* increased the pairwise genetic distance between accessions to 0.14 (between 127 accessions of *A. clauda* and 169 accessions of other species, Table S3). The genetic distance computed between species varied from 0.006 between *A. wiestii* and *A. atlantica* to 0.020 between *A. canariensis* and *A. prostrata* (Table 3). A search for shared haplotypes among the sequences of the 169 accessions identified 156 haplotypes of which seven were common up to five accessions that belong to different species (Table S4). One of the haplotypes was common among five accessions belonging to *A. canariensis* (OM004580), *A. longiglumis* (OM004605), *A. wiestii* (OM004652), *A. hirtula* (OM004673), and *A. atlantica* (OM004718). The second shared haplotype was observed in four accessions that belong to *A. prostrata* (OM004624), *A. wiestii* (OM004653), *A. hirtula* (OM004669), and *A. damascena* (OM004733). The third shared haplotype was common in three accessions that belong to *A. wiestii* (OM004651), *A. hirtula* (OM004677), and *A. atlantica* (OM004717). The remaining four haplotypes were common between *A. wiestii* (OM004656) and *A. hirtula* (OM004687), between *A. wiestii* (OM004661) and *A. atlantica* (OM004722), between *A. wiestii* (OM004665) and *A. atlantica* (OM004727), and between *A. wiestii* (OM004666) and *A. hirtula* (OM004693). *A. clauda* (previously defined as a C-genome species) had a haplotype (OK273996) that was shared with five accessions belonging to *A. canariensis* (OM004580), *A. longiglumis* (OM004605), *A. wiestii* (OM004652), *A. hirtula* (OM004673), and *A. atlantica* (OM004718). The frequency of each haplotype ranged from 0.006 to 0.030.

As shown in Table 4, differences in species accounted for 27.2% of the molecular variation, and the remaining 72.8% of the variation was observed among accessions within each species. The proportion of molecular variance among species that belong to the same genome was computed only for the three As-genome species (*A. atlantica*, *A. hirtula*, and *A. wiestii*), which accounted for 6.9% of the total variance. Of the 21 pairwise comparisons of F_ST_ values among the eight species (Table 5), we observed moderate genetic differentiation between *A. wiestii* and *A. atlantica* (0.065) and between *A. wiestii* and *A. hirtula*. *A. canariensis* showed moderate genetic differentiation with *A. damascena*, *A. longiglumis*, *A. atlantica*, and *A. wiestii*, while *A. longiglumis* showed great genetic differentiation with *A. atlantica*, *A. hirtula*, and *A. wiestii*. Similarly, great genetic differentiation was observed between *A. damascena* and *A. prostrata* plus and between *A. atlantica* and *A. hirtula*. The remaining ten pairs of F_ST_ values ranged from 0.273 to 0.452, which indicated very great genetic differentiation. *A. clauda* (species with both C- and A-genome ribotypes) expectedly showed very great genetic differentiation but in the range of all studied species (from 0.311 to 0.376).

## 4. Discussion

Using 18S rDNA, ITS1, and 5.8S rDNA sequences of 169 accessions from seven species, we have shown substantial intragenome polymorphisms in rDNA. The level of intragenome variation differed among species, which was evident from differences in the number of ribotypes, haplotypes, nucleotide diversity indices, genetic distance, and genetic differentiation. For example, we found a single ribotype for 15 accessions of *A. longiglumis* and four ribotypes among 17 accessions of *A. wiestii* (Table 1). Both species were represented by a similar sample size but differed in the number of ribotypes.

Among them, there are species with one, two, or more main ribotypes. The intragenomic heterogeneity of 35S rDNA in allopolyploids is usually explained by the fact that many plant species, primarily allopolyploids, arose due to interspecific hybridization. Despite the processes of homogenization (concerted evolution) of rDNA repeats, allopolyploids can retain a part of the rDNA of both paternal ancestors for some time [55,56,57,58,59]. In addition, a high level of SNPs in DNA may be an effect of genomic shock accompanying interspecies hybridization [60,61]. Cases of abnormally high rDNA polymorphism that were possibly associated with the instability of the hybrid genome are known [34,56,62].

Possible causes of intragenomic rDNA polymorphism in diploid *Avena* species are shown in Figure 4. Parent species (A and B) may have different sets of ribotypes (Figure 4). Diploid *Avena* species with the A-genome usually have two nucleolar organizers per haploid genome [7,8,32,63,64], sometimes three [8]. In the present study, we found two related ribotypes (Ac1 and Ac2) for the Ac-genome *A. canariensis* species which agree with the two nucleolar organizer regions (NORs) reported in the haploid chromosomes of the same species [8,32,64]. *A. damascena* (Ad genome) has been found to possess two major NORs [7,8,32,64] which also agree with the two ribotypes (Ad1 and Ad2/As3) that we identified in the current study. The same was true for *A. atlantica* and *A. hirtula*, which possess the As genome. The fact that we saw two main ribotypes in several diploid *Avena* species (Table 1) may be related to the features of homogenization processes. The most likely explanation for this finding is that intra-NOR homogenization events occur at much higher rates than homogenization between NORs located on different chromosomes [57,65].

**Figure 6:**
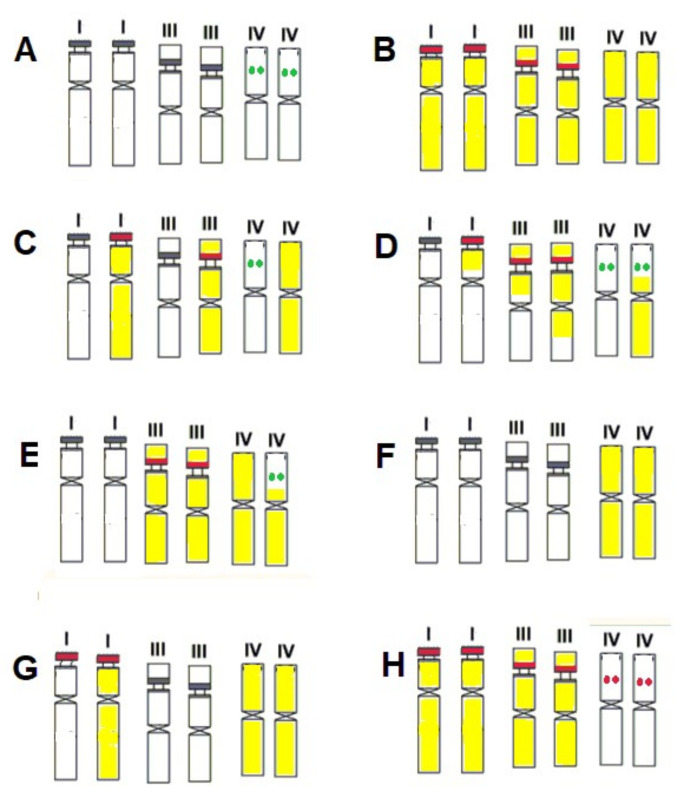
Possible causes of intragenomic rDNA heterogeneity in *Avena* with A genomes (only chromosomes carrying major and minor NORs are shown [7]). (**A**) Diploid species have two major and one minor NORs (as in *A. damascena* [8]). The rDNA of both main NORs belong to the same ribotype as a result of concerted evolution. In the minor locus, 35S rDNA belongs to another ribotype. (**B**) A diploid having a single main ribotype, like *A. longiglumis*. (**C**) F1 hybrid between species A and B. (**D**–**F**) Chromosomes of Fn hybrids—chromosomes after recombination (**D**,**E**) or without recombination (**F**). (**G**) Diploid hybrids with different chromosome sets as a result of selection pressure. (**H**) rDNA loci of the minor site are homogenized towards the major ribotype. This figure is published with the kind permission of E. D. Badaeva and colleagues. It is reproduced from [7], with modifications.

Different ribotypes may be located on different chromosomes (Figure 6) because these chromosomes can have different origins (e.g., homoploid hybrid genome from different ancestors). In the modern cultivated barley genome, for example, chromosomes 1–3 and chromosomes 4–7 originated from two different wild-barley populations, with the largest chromosomes from the Near East Fertile Crescent and the smallest chromosomes from the Tibetan Plateau [66].

Cytogenetic examination shows that the three diploid As-genome oat species (*A. hirtula*, *A. wiestii*, and *A. atlantica*) are highly similar in karyotype structure and chromosome C-banding patterns [8], although their hybrids are interfertile [3,20,21,22]. In the present study, these three species had three common ribotypes (Table 1) and two to five common haplotypes (Table S4), which were likely the main reasons that contributed to the low genetic distances (0.006–0.010) and moderate to great genetic differentiation (0.065–0.172) observed among a pair of these species. The three species were also very close in both the phylogenetic tree and multidimensional scaling (Figure 3 and Figure 4). However, in addition, the *A. wiestii* genome contained the Ad2/As3 ribotype, and this showed us that *A. wiestii* was related to *A. hirtula* and to *A. damascena* (Ad).

The genome of *Avena prostrata* and *A. canariensis* contains a minor fraction of the Ad2/As3-ribotype, which can be considered either as a trace of the ancestral genome or as a result of introgression after crossing with Ad or Ad-species. It has been shown that *A. prostrata* can be crossed experimentally with *A. longiglumis* to produce hybrids [67] which can produce partially fertile hybrids in crosses with *A. canariensis* [68]. *Avena canariensis* is endemic to the Canary Islands and occurs only on Fuerteventura and Lanzarote islands, which are close to two islands that are close to Morocco [3]. Therefore, the lack of common ribotypes and haplotypes between these species was not surprising.

All attempts to hybridize *A. longiglumis* with the members of the As group have thus far failed due to sexual isolation [20,22,68]. Cytological studies conducted by Rajhathy [68], Thomas and Jones [21], and Ladizinsky [66] demonstrated the presence of pronounced structural differences between the karyotype of *A. longiglumis* and As-genome oats. Although we observed at least one shared ribotype and haplotype between *A. longiglumis* and the other three As-species, these four species are quite genetically different, which is obvious from the pairwise F_ST_ values (Table 5), NT trees, and the MDS plots (Figure 3 and Figure 4). The reproductive isolation data reviewed by Loskutov [23] suggest that As oats are actually homoploid hybrids, whose ancestral species is *A. longiglumis*. The act of interspecific hybridization between *A. longiglumis* and the ancestors of As-oats could stimulate a saltational rearrangement of the karyotype, a series of chromosomal interchanges affecting all chromosomes. We emphasize that it is the As-karyotype that is rearranged, since Ap and Al genomes exhibit remarkable affinity of their chromosome architecture [66].

Our previous studies have revealed unexpectedly high rDNA heterogeneity in *A. clauda*, which belongs to the C-genome group [3,4]. In the present study, we found out that ribotypes Cc2A (#19), Cc2B (#20), and Cc2C (#21) in *A. clauda* (Table 1 and Figure 3) were specific variants of the A-genome ribotype family. In particular, the ribotype Cc2A was closely related to the ribotypes As1 (#9 and 13) and As5 (#11), which suggested that *A. clauda* may be a hybrid between As- and C-genome oats. A previous study reported two main and two minor NORs in the *A. clauda* haploid genome [7]. Different ribotype families may be located in different NORs in this species.

*F_ST_* values are indicative of the evolutionary processes that influence the extent of genetic divergence among species, populations, or groups, with <0.05 indicating little, 0.05–0.15 moderate, 0.15–0.25 great, and >0.25 very great genetic differentiation [69]. In the present study, most pairs of species showed either great or very great genetic differentiation (Table 5). Such results are expected in oat due to the reproductive isolation reported by several studies for multiple reasons, including chromosomal rearrangements, the relocation of 5S and 45S rDNA loci, changes in the sequences of rDNA [5,7,8,70,71,72], and major cytological differences in repetitive DNA content [70]. Thus, despite the fact that all species of the genus *Avena* studied by us were diploids, we could see that most of them contained several different rDNA families. A comparative study of rDNA patterns in individual species shows that the rDNA pattern is, as a rule, mosaic and, in all cases, species-specific. We can suppose that the *Avena* species, carriers of the Al, Ap, and As genomes, are, in fact, not primary diploids reproductively isolated from each other, but some kind of a Mediterranean introgressive hybridization species complex [73], sporadically entering into interspecific hybridization. At the same time, A-genome oat species can reflect the hybridization events that occurred in their evolutionary past as the way of their speciation.

## Figures and Tables

**Table 1:** Summary of the oat species used in the present study and their genome type and ribotypes. *A. clauda* (C genome) was also used in the analysis because it had some ribotypes belonging to the A genome in its rDNA pool.

Species	Sample ID	Country of Origin	Accession Number	Number of Accessions	Genebank	Genome	Number of NORs in Haploid Genome	Total Number of Reads	Ribotype Number	Ribotype Symbol	Number of Reads	% From the Total Number of the Reads
*Avena canariensis*	k-2114	Spain	From OM004567 to OM004604	38	A. Diederichsen	Ac	2 [1–3]	20606	1	Ac1	10,050	49
									2	Ac2	1908	9
*Avena damascena*	k-1862	Syria	From OM004732 to OM004735	4		Ad	2-3 [1–3]	12296	3	Ad1	4910	40
									4	Ad2/As3	3936	32
*Avena longiglumis*	k-1881	USA	From OM004605 to OM004619	15		Al	2 [1–3]	12736	5	Al1/As2	9412	74
*Avena prostrata*	k-2055	Spain	From OM004620 to OM004650	31		Ap	2 [1]	17690	6	Ap1	4933	28
									7	Ap2	3188	18
									8	Ap3	2559	15
*Avena atlantica*	k-2108	Morocco	From OM004717 to OM004731	15	A. Diederichsen	As	2 [1]	16241	9	As1	6353	39
									10	Al1/As2	4571	28
*Avena hirtula*	k-1878	Spain	From OM004668 to OM004716	49		As	2 [4]	16538	11	As5	12,605	40
									12	Ad2/As3	3587	11
*Avena wiestii*	k-2119	Israel	From OM004651 to OM004667	17	A. Diederichsen	As	2 [1,4]	16725	13	As1	4005	24
									14	Al1/As2	2901	17
									15	Ad2/As3	2598	16
									16	As4	1974	12
*Avena clauda*	k-269	Azerbaijan	From OK273905 to OK274031	127	V. N. Soldatov	Cc	2-4 [3–5]	40168	17	Ccc1A	5320	13
									18	Cc1B	2695	7
									19	Cc2A	3195	8
									20	Cc2B	1777	4
									21	Cc2C	1305	3

**Table 2:** Summary of diversity indices for accessions belonging to each species and all species.

Samples	N	S	P_s_	Θ	π	D
All	296	109	0.318	0.051	0.031	−2.291
A-genomes	169	92	0.269	0.047	0.013	−2.291
As-genomes	81	38	0.111	0.022	0.009	−1.870
*Avena canariensis* [Ac]	38	40	0.117	0.028	0.008	−2.476
*Avena longiglumis* [Al]	15	11	0.032	0.010	0.005	−1.901
*Avena prostrata* [Ap]	31	34	0.099	0.025	0.014	−1.561
*Avena wiestii* [As]	17	11	0.032	0.010	0.007	−1.145
*Avena hirtula* [As]	49	23	0.067	0.015	0.010	−1.040
*Avena atlantica* [As]	15	11	0.032	0.010	0.005	−1.955
*Avena damascena* [Ad]	4	3	0.009	0.005	0.004	−0.754
*Avena clauda* [Cc]	127	34	0.099	0.018	0.037	3.160

**Table 3:** Pairwise mean genetic distance between species.

Species	No. of Accessions	*A. canariensis*	*A. longiglumis*	*A. prostrata*	*A. wiestii*	*A. hirtula*	*A. atlantica*	*A. damascena*
A. canariensis	38							
A. longiglumis	15	0.010						
A. prostrata	31	0.020	0.015					
A. wiestii	17	0.012	0.007	0.016				
A. hirtula	49	0.015	0.010	0.018	0.010			
A. atlantica	15	0.012	0.007	0.017	0.006	0.010		
A. damascena	4	0.014	0.009	0.014	0.010	0.012	0.011	
A. clauda	127	0.051	0.043	0.051	0.044	0.045	0.045	0.046

**Table 4:** Analysis of molecular variance (AMOVA) for the extraction of sequence variation among and within species.

Source of Variation	Degree of Freedom	Sum of Squares	Variance Components	Percentage Variation	F_ST_
Among species	6	125.46	1.00	27.24	0.27
Within species	162	438.91	2.68	72.76	
Total	168	564.36	3.68		
Among genomes	4	125.46	0.80	21.94	0.29
Among species within As-genome	2	16.58	0.25	6.89	
Within species	162	422.33	2.61	71.17	
Total	168	564.36	3.66		

**Table 5:** Pairwise FST between pairs of eight oat species.

Species	*A. canariensis*	*A. damascena*	*A. longiglumis*	*A. prostrata*	*A. atlantica*	*A. hirtula*	*A. wiestii*
*A. canariensis*							
*A. damascena*	0.193						
*A. longiglumis*	0.167	0.452					
*A. prostrata*	0.310	0.186	0.295				
*A. atlantica*	0.208	0.273	0.183	0.326			
*A. hirtula*	0.289	0.278	0.199	0.323	0.172		
*A. wiestii*	0.218	0.414	0.178	0.315	0.065	0.111	
*A. clauda*	0.360	0.311	0.334	0.376	0.354	0.366	0.340

## Data Availability

All supplementary materials are available on https://susy.mdpi.com/user/manuscripts/displayFile/48009b36d3716bf9556b62f7edb8b385/supplementary.

## Supplementary Materials

The following are available online at https://www.mdpi.com/article/10.3390/plants11091103/s1, Table S1, Sequences of 296 oat accessions; Table S2, Genetic distances and multidimensional scaling of A-genome species (*Avena clauda* is not included); Figure S1, Neighbor joining tree and multidimensional scaling based on sequences of 169 accessions from seven Avena species. Each species was represented by 4-49 accessions; Table S3, Pairwise distances between accessions of the A-genome species and C-genome species *Avena clauda*; Table S4, Summary of the 156 haplotypes obtained from sequences of 169 oat accessions.
