# Supplementary material for: New Insights into the Genomic Structure of Avena L.: Comparison of the Divergence of A-Genome and One C-Genome Oat Species
Source: Plants (Basel). 2022 Apr 19;11(9):1103. doi: 10.3390/plants11091103 (PMC9102028; doi:10.3390/plants11091103)
Supplement: Supplementary file 1 [file plants-11-01103-s001.zip › Supplementary/Figure S1 NJ tree and MDS.pptx]

## Slide 1
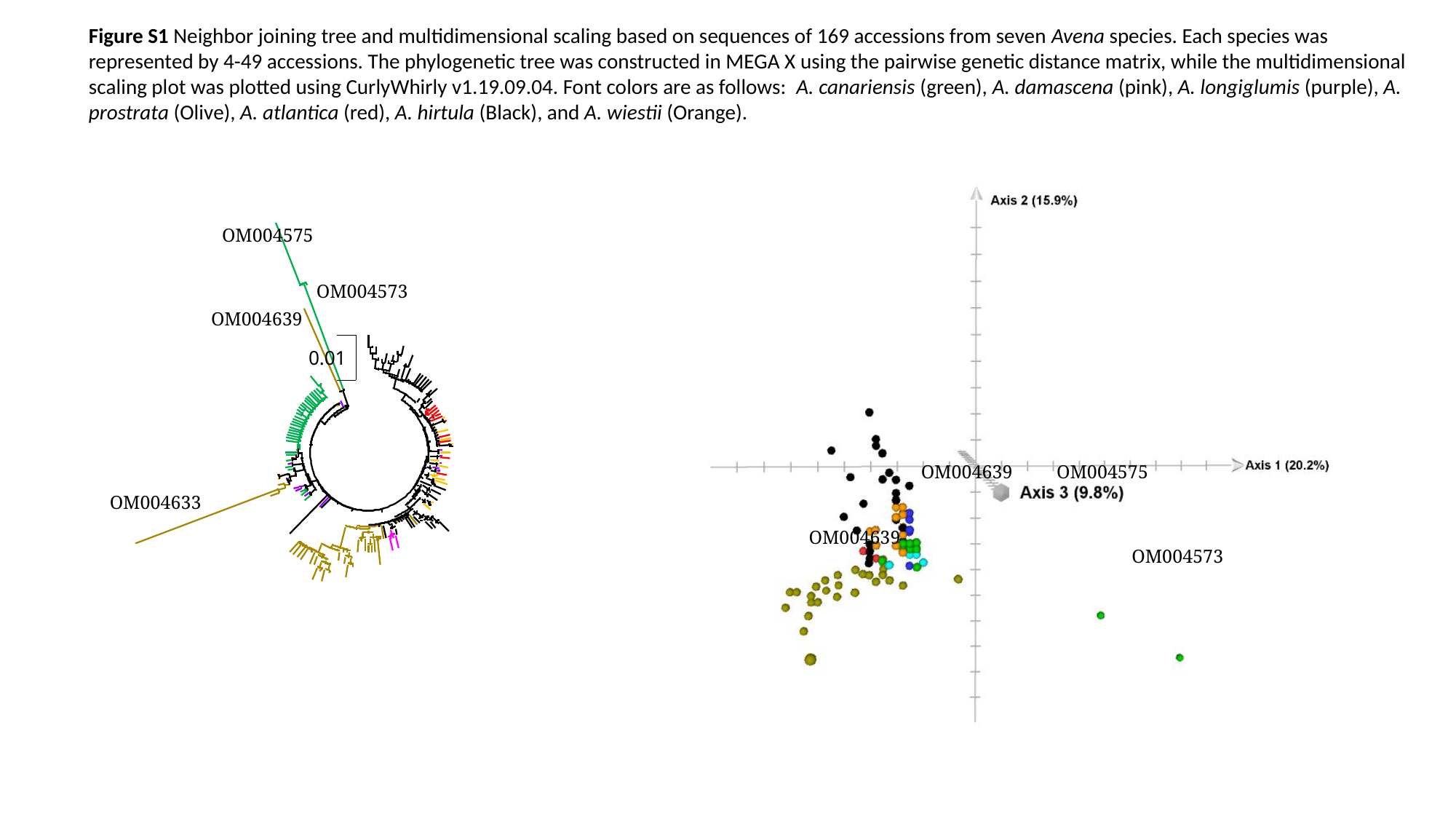

Figure S1 Neighbor joining tree and multidimensional scaling based on sequences of 169 accessions from seven Avena species. Each species was represented by 4-49 accessions. The phylogenetic tree was constructed in MEGA X using the pairwise genetic distance matrix, while the multidimensional scaling plot was plotted using CurlyWhirly v1.19.09.04. Font colors are as follows: A. canariensis (green), A. damascena (pink), A. longiglumis (purple), A. prostrata (Olive), A. atlantica (red), A. hirtula (Black), and A. wiestii (Orange).
OM004639
OM004575
OM004639
OM004573
OM004575
OM004573
OM004639
OM004633
